# Supplementary material for: The Predictive Impact of HPV Genotypes, Tumor Suppressors and Local Immune Response in the Regression of Cervical Intraepithelial Neoplasia 2-3: A Prospective Population-Based Cohort Study
Source: Int J Mol Sci. 2025 May 28;26(11):5205. doi: 10.3390/ijms26115205 (PMC12155477; doi:10.3390/ijms26115205)
Supplement: Supplementary file 1 [file ijms-26-05205-s001.zip › ijms-3646962-supplementary.pdf]

Supplementary table S1. Regression status (yes or no), regression rate per 1000 persons, and hazard ratio (HR) according to CIN2-3 lesions infected with only one HPV genotype.

**Variables**	**Regression yes/no**	**Regression rate Per 1000**	**HR (95% CI)**
**High-regression group[1]**			
**No**	12/56	1.53 (0.74 – 2.32)	1.00
**Yes**	21/13	4.46 (2.63 – 6.30)	2.93 (1.51 – 5.65)
**Low-regression group[2]**			
**No**	15/23	3.56 (2.14 – 4.98)	1.00
**Yes**	9/46	1.42 (0.56 – 2.28)	0.40 (0.19 – 0.82)

[1] HPVs 18, 45, 33, 35, 39.

[2] HPVs 16, 31, 52, 58.

Supplementary table S2. Biomarkers and regression

Variables	Regression y/n	HR (95% CI)[1]
CD4 epithelial		
≤ 6	10/50	1.00
> 6	24/78	1.41 (0.67 – 2.97)
CD4 stroma		
≤ 195	33/103	1.00
> 195	1/25	0.19 (0.03 – 1.41)
CD8 epithelial		
≤ 30	23/100	1.00
> 30	11/28	1.23 (0.61 – 2.48)
CD8 stroma		
≤ 201	11/61	1.00
> 201	22/67	1.68 (0.81 – 3.47)
CD25 epithelial		
≤ 5	14/73	1.00
> 5	20/55	1.37 (0.69 – 2.71)
CD25 stroma		
≤ 25	7/41	1.00
> 25	26/86	1.43 (0.61 – 3.36)
CD138 stroma		
≤ 40	18/53	1.00
> 40	15/75	0.72 (0.37 – 1.39)
Ki67 SI		
≤ 0.81	26/67	1.00
> 0.81	6/54	0.35 (0.14 – 0.86)
Ki67 pos ncl		
≤ 0.81	12/21	1.00
> 0.81	20/100	0.61 (0.28 – 1.31)
DC4/CD25 in stroma		
≤ 9.75	26/116	1.00
> 9.75	7/11	2.70 (1.23 – 5.92)
DC4/CD8 in stroma		
≤ 0.67	27/81	1.00
> 0.67	6/47	0.43 (0.18 – 1.07)
DC4/CD8 in epithelial		
≤ 0.77	19/89	1.00

> 0.77	15/39	1.62 (0.83 – 3.14)
DC25/CD8 in stroma		
≤ 0.191	18/61	
> 0.191	15/66	0.65 (0.32 – 1.33)
DC25/CD8 in epithelial		
≤ 0.330	27/98	
> 0.330	6/29	0.73 (0.31 – 1.72)

[1] Hazard ratios (HR) and 95% confidence intervals (CI) was estimated using Cox proportional hazards ration model
